# Genome sequence of a high agarase-producing strain *Flammeovirga* sp. SJP92

**DOI:** 10.1186/s40793-017-0221-y

**Published:** 2017-01-26

**Authors:** Qi Dong, Lingwei Ruan, Hong Shi

**Affiliations:** 1 State Key Laboratory Breeding Base of Marine Genetic Resources, No. 184 Daxue Road, Xiamen, Fujian People’s Republic of China; 2 Key Laboratory of Marine Genetic Resources of State Oceanic Administration, Third Institute of Oceanography, State Oceanic Administration, No. 184 Daxue Road, Xiamen, Fujian People’s Republic of China; 3 Key Laboratory of Marine Genetic Resources of Fujian Province, No. 184 Daxue Road, Xiamen, Fujian People’s Republic of China; 4 South China Sea Bio-Resource Exploitation and Utilization Collaborative Innovation Center, No. 184 Daxue Road, Xiamen, Fujian People’s Republic of China

**Keywords:** *Flammeovirga*, Genome, High agarase-producing

## Abstract

*Flammeovirga* sp. SJP92 is a Gram-negative, aerobic, rod-shaped, non-motile and non-flagellated strain that belongs to the family *Flammeovirgaceae* of the class *Cytophagia*. The strain was isolated from the intestine of abalone, which produces many extracellular agarases and exhibits efficient degradation activities on various polysaccharides, especially agarose. Here we present the high-quality draft genome of *Flammeovirga* sp. SJP92, together with its phenotypic characteristics. The genome sequence is 8, 534, 834 bp, which comprised with one chromosome and no plasmid. It contained 6, 291 protein-coding and 99 RNA genes, including 93 tRNA, 5 rRNA and 1 ncRNA genes.

## Introduction

*Flammeovirga* is one of genera belonging to the family *Flammeovirgaceae* of the class *Cytophagia*. There are five species have been reported in this genus, including *F. aprica* [1], *F. arenaria* *,* *F. yaeyamensis* [2], *F. kamogawensis* [3] and *F. pacifica* [4]. They are all marine bacterium and have a potent ability to degrade marine complex polysaccharides, such as agar, carrageenan [3, 5–8]. Among them, only two draft genome sequences have been published [9], namely *Flammeovirga* sp. OC4 (NZ_JTAM01000001.1) [5] and *F. pacifica* WPAGA1^T^ (=CCTCC AB 2010364T=LMG 26175T=DSM 24597T=MCCC 1A06425T) [7].

*Flammeovirga* sp. SJP92 with high-producing agarase was isolated and identified from the intestine of abalone in Xiamen, China. It is closely related with *Flammeovirga* sp. NBRC 100896 (AB681288.1) and shared 99% similarities of 16S rRNA. In order to provide more genome information of *Flammeovirga* species and realize the function of *Flammeovirga* sp. SJP92 when degradingmarine complex polysaccharides, the genome of *Flammeovirga* sp. SJP92 was sequenced. In this study, we summarized its genomic characteristics, as well as general phenotypic properties. Other species of *Flammeovirga* genus were also compared with *Flammeovirga* sp. SJP92 in both phenotypic and genomic aspects.

## Organism information

### Classification and features

**Fig. 1:**
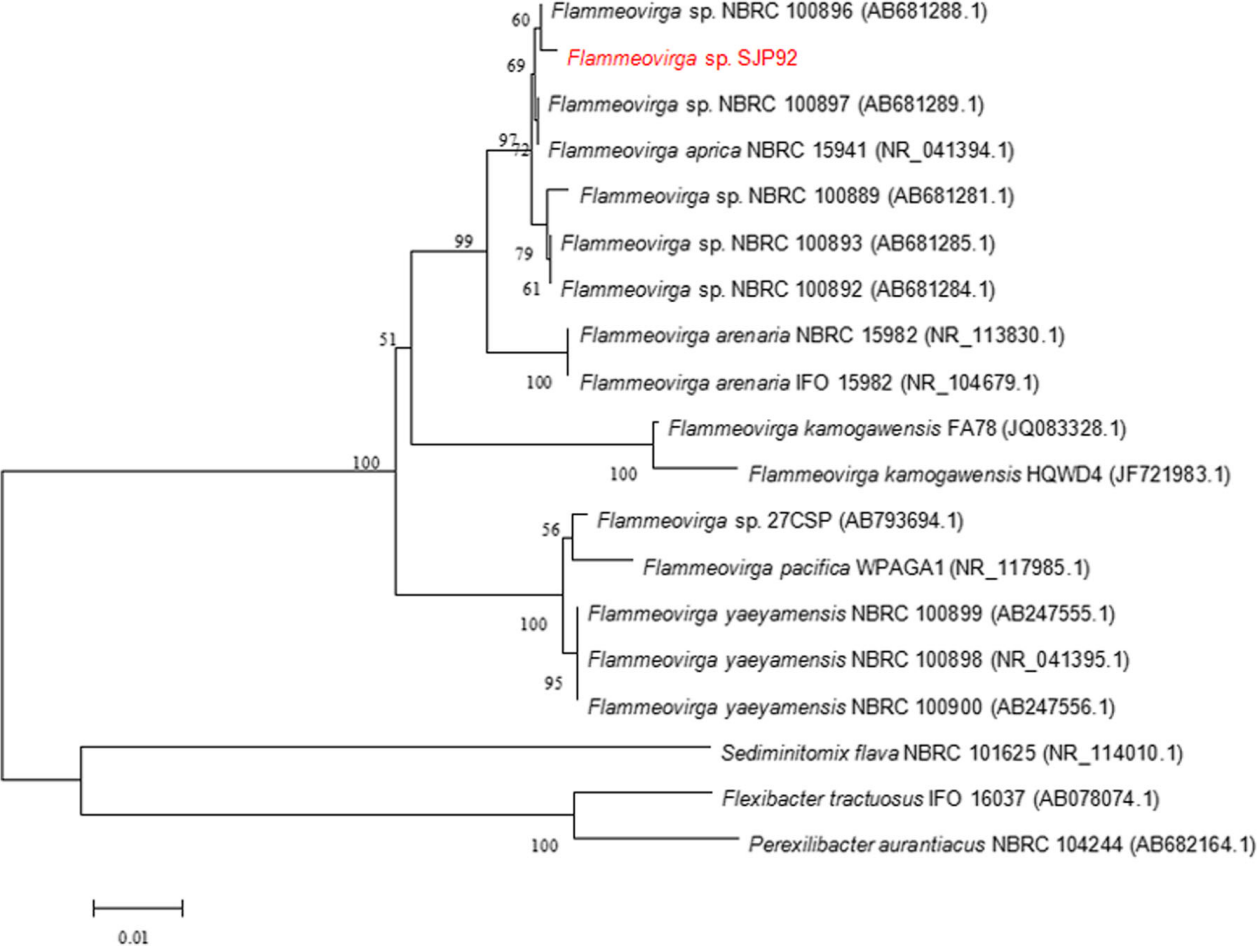
Phylogenetic tree highlighting the position of *Flammeovirga* sp. SJP92 relative to other type and non-type strains with finished or non-contiguous finished genome sequences within the family *Flammeovirga*. Accession numbers of 16S rRNA gene sequences are indicated in brackets. Sequences were aligned using ClustalX [14] and a neighbor-joining tree obtained using the maximum-likelihood method within the MEGA version4.0 [20]. Numbers adjacent to the branches represent percentage bootstrap values based on 1000 replicates

**Fig. 2:**
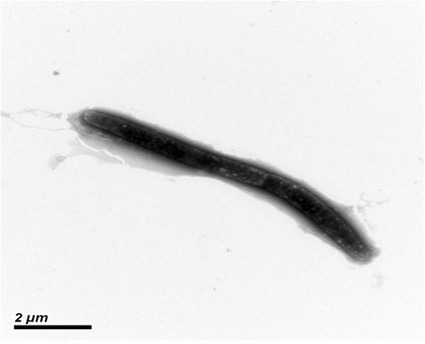
Transmission electron micrograph of *Flammeovirga* sp. SJP92, using a JEM-100CX at an operating voltage of 120 KV. The scale bar represents 2 μm

*Flammeovirga* sp. SJP92 was isolated from the digestion guts of abalone with high agar-degrading ability, and deposited in China General Microbiological Culture Collection Center (CGMCC 10071). Based on the phylogenetic tree constructed with 16S rRNA, *Flammeovirga* sp. SJP92 is closely related with *Flammeovirga* sp. NBRC 100896 (AB681288.1) (Fig. 1). It is Gram-negative, curved-rods (0.75 μm wide and 11–13 μm long) after growth on 2216E plate for 3 days at 30 °C. It is aerobic and not motile without any flagella (Fig. 2). Also it is able to utilize a relatively wide spectrum of carbon substrates for growth, including agar, starch, carrageenan, L-fructose, Tween40, Tween80, galactose, lactose and so on, but it cannot utilize cellulose. Its growth temperature ranges from 15 to 40 °C with optimum between 25 and 30 °C. In addition, the optimum salinities for the growth of *Flammeovirga* sp. SJP92 were 2 ~ 4% (Table 1). When compared with other *Flammeovirga* species, this strain is different from *F. pacifica* WPAGA1^T^ [8] and *F. aprica* NBRC 15941 T [2] in catalase, urease and esterase lipase and in the utilization of starch, D-Mannitol, L-fructose, Tween40&80 and D-xylose, differences were also observed in growth temperature range (Table 2).

**Table 1:** Classification and general features of Flammeovirga sp.SJP92

MIGS ID	Property	Term	Evidence Code^a^
	Current classification	Domain *Bacteria*	TAS [21]
		Phylum *Bacteroidetes*	TAS [22]
		Class *Cytophagia*	TAS [23, 24]
		Order *Cytophagales*	TAS [25, 26]
		Family *Flammeovirgaceae*	TAS [27]
		Genus *Flammeovirga*	TAS [1]
		Species *Flammeovirga* sp.	TAS [5, 7]
		Strain SJP92	IDA
	Gram Stain	Negative	IDA
	Cell shape	Curved-rods	IDA
	Motility	None	IDA
	Sporulation	Non-sporulating	IDA
	Temperature range	15 ~ 40 °C	IDA
	Optimum temperature	25 ~ 30 °C	IDA
	pH range; Optimum	5 ~ 9, 8	IDA
	Carbon source	Agar, Starch, Carrageenan, D-galactose, L-fructose, Tween40&80	IDA
MIGS-6	Habitat	Intestinal tract	IDA
MIGS-6.3	Salinity	0.5–8% NaCl (w/v)	IDA
MIGS-22	Oxygen	Aerobic	IDA
MIGS-15	Biotic relationship	Free-living	IDA
MIGS-14	Pathogenicity	Unknown	NAS
MIGS-4	Geographic location	Xamen city, China	IDA
MIGS-5	Sample collection	October 2006	IDA
MIGS-4.1	Latitude	24°26'	IDA
MIGS-4.2	Longitude	118°04'	IDA
MIGS-4.4	Altitude	Sea level	IDA

^a^Evidence codes: *IDA* Inferred from Direct Assay, *TAS* Traceable Author Statement (i.e., a direct report exists in the literature), *NAS* Non-traceable Author Statement (i.e., not directly observed for the living, isolated sample, but based on a generally accepted property for the species, or anecdotal evidence). These evidence codes are from the Gene Ontology project [28]. If the evidence code is IDA, then the property should have been directly observed for a live isolate by one of the authors, or an expert or reputable institution mentioned in the acknowledgement

**Table 2:** Differential phenotypic characteristics between *Flammeovirga* sp. SJP92 and other *Flammeovirga* species

Characteristic	1	2	3
Cell diameter (um)	11 ~ 13 × 0.75	3.0 ~ 8.0 × 0.5 ~ 0.8	1.7 ~ 96 × 0.5 ~ 0.9
Salinity/Optimum(w/v)	0.5 ~ 8%/2 ~ 4%	0–5%/3%	1–5%/3%
Temperature range (°C)	15 ~ 40	4–42	15–30
Number of polar flagella	None	None	None
Production of			
Agarase	+	+	+
Catalase	+	−	−
Oxidase	+	+	+
Esterase lipase	_	±	±
Urease	+	−	−
β-Galactosidase	+	±	ND
α-Galactosidase	+	+	ND
Nitrate reductase	+	+	+
Alkaline/Acid phosphatase	+	+	+
Carbon source			
Gelatin	ND	−	−
Agar	+	+	+
Starch	+	−	−
Cellulose	−	−	−
D-galactose	+	+	+
D-Mannitol	−	±	−
L-fructose	+	+	−
Tween40&80	+	−	−
D-xylose	-	+	+
Geographic location	XiaMen, China	157 °249′ 310″ E 19° 309′ 300″ N	Iriomote/Ishigaki Islands
Habitat	Intestinal tract	Deep-sea sediment	Seaweeds/coastal sands/dead leaves

Strains: 1, *Flammeovirga* sp. SJP92; 2, *F. pacifica* WPAGA1^T^; 3, *F. aprica* NBRC15941^T^.+: positive result, −: negative result, ±:weak positive result, *ND* no data available

## Genome sequencing information

### Genome project history

This organism was initially selected for sequencing on the basis of its high agar-degrading ability. Sequencing of the *Flammeovirga* sp. SJP92 genome was performed at the Beijing Novogene Bioinformatics Technology Co., Ltd. The Whole Genome Shotgun project has been deposited at the DDBJ/EMBL/GenBank database under the accession number LQAQ00000000. The project information and its association with MIGS version 2.0 compliance were presented in Table 3 [9].

**Table 3:** Genome sequencing project information for *Flammeovirga* sp. SJP92

MIGS ID	Property	Term
MIGS-31	Finishing quality	High-quality draft
MIGS-28	Libraries used	500 bp pair-end&5 kb mate-end libraries
MIGS-29	Sequencing platforms	Illumina HiSeq2500,
MIGS-31.2	Fold coverage	215×
MIGS-30	Assemblers	SOAPdenovo v.2.04
MIGS-32	Gene calling method	NCBI PGAP pipeline
	Locus Tag	AVL50
	GenBank ID	LQAQ00000000
	GenBank Date of Release	March 9th, 2016
	GOLD ID	NA
	BIOPROJECT	PRJNA306821
MIGS-13	Source Material identifier	SJP92
	Project relevance	Agriculture, industry

### Growth conditions and genomic DNA preparation

*Flammeovirga* sp. SJP92 was incubated aerobically in the modified 2216E medium (2.2% NaCl, 0.365% MgCl_2·_6H_2_O, 0.729% MgSO_4_ · 7H_2_O, 0.03% CaCl_2_ · 2H_2_O, 0.05% KCl, 0.042% KH_2_PO_4_, 0.005% NaBr, 0.002% SrCl · 6H_2_O, 0.002% Fe (NH_4)_ Citrate, 1.326% tryptone) supplied with 0.2% agar. After incubation at 32 °C, 200 rpm for 24 h, the bacteria was collected at 13000 rpm for 30–60 min at 4 °C. The CTAB/NaCl method [10] was used for the extraction of chromosomal DNA of *Flammeovirga* sp. SJP92.

### Genome sequencing and assembly

The genome of *Flammeovirga* sp. SJP92 was sequenced with MPS (massively parallel sequencing) Illumina technology. Three DNA libraries were constructed: a paired-end library with an insert size of 500 bp and two mate-pair libraries with an insert size of 5 kb. The 500 bp library and the 5 kb libraries were sequenced using an Illumina HiSeq2500 by PE125 strategy. Library construction and sequencing was performed at the Beijing Novogene Bioinformatics Technology Co., Ltd. Quality control of both paired-end and mate-pair reads were performed using in-house program. The final coverage reached 215-folds of the genome. SOAPdenovo [11, 12] was used for sequence assembly, and the final assembly yielded 123 contigs which generated a genome of 8.53 Mb.

### Genome annotation

The genes of *Flammeovirga* sp. SJP92 was identified by NCBI Prokaryotic Genome Annotation Pipeline server online [13]. Functional predicted was performed by comparing them with sequences in RPS-BLAST against Clusters of Orthologous Groups database and pfam database [14–16]. SignalP was used to predict signal peptide [17], and transmembrane helice was analyzed by TMHMM program [18]. CRISPRFinder was used for CRISPR identification [19].

## Genome properties

The *Flammeovirga* sp. SJP92 genome has only one circular chromosome of a total size of about 8, 534, 834 bp with a 34.80% GC content (containing 123 contigs, 44 scaffolds).6519 genes were predicted, of which 6291 genes were protein-coding genes. 2660 genes (40.8%) were assigned to putative function and annotated as hypothetical proteins. And 99 RNAs (including 93 tRNAs, 5 rRNAs and 1 ncRNA), 127 pseudo genes were also identified. The properties and the statistics of the genome were summarized in Table 4, and Table 5 presented the distribution of genes into COGs functional categories. 3752 genes (57.55%) were assigned to COG functional categories, the most abundant COG category was “General function prediction only” (561 proteins) followed by “Signal transduction mechanisms” (401 proteins), “Transcription” (382 proteins), “Function unknown” (350 proteins), “Cell wall/membrane/envelope biogenesis” (347 proteins), “Inorganic ion transport and metabolism” (318 proteins), and “Carbohydrate transport and metabolism” (306 proteins).

**Table 4:** Genome Statistics for *Flammeovirga* sp. SJP92

Attribute	Value	% of Total^a^
Genome size (bp)	8,534,834	100.0
DNA coding (bp)	7,309,656	85.64
DNA G + C (bp)	2,970,122	34.80
DNA scaffolds	44	100.00
Total genes	6519	100.00
Protein-coding genes	6291	96.5
RNA genes	99	1.52
Pseudo genes	127	1.95
Genes in internal clusters	NA	NA
Genes with function prediction	4240	65.04
Genes assigned to COGs	3752	57.55
Genes assigned Pfam domains	3964	60.81
Genes with signal peptides	1658	25.43
Genes with transmembrane helices	1510	23.16
CRISPR repeats	1	0.01

^a^The total is based on either the size of the genome in base pairs or on the total number of protein coding genes in the annotated genome

*NA* not available

**Table 5:** Number of protein coding gene of *Flammeovirga* sp. SJP92 associated with COG functional categories

Code	value	% age	Description
J	178	2.83	Translation, ribosomal structure and biogenesis
A	0	0	RNA processing and modification
K	382	6.07	Transcription
L	199	3.16	Replication, recombination and repair
B	2	0.03	Chromatin structure and dynamics
D	47	0.75	Cell cycle control, cell division, chromosome partitioning
V	90	1.43	Defense mechanisms
T	401	6.37	Signal transduction mechanisms
M	347	5.51	Cell wall/membrane/envelope biogenesis
N	34	0.54	Cell motility
U	80	1.27	Intracellular trafficking, secretion, and vesicular transport
O	158	2.51	Posttranslational modification, protein turnover, chaperones
C	215	3.42	Energy production and conversion
G	306	4.8	Carbohydrate transport and metabolism
E	269	4.23	Amino acid transport and metabolism
F	86	1.37	Nucleotide transport and metabolism
H	193	3.06	Coenzyme transport and metabolism
I	147	2.34	Lipid transport and metabolism
P	318	5.05	Inorganic ion transport and metabolism
Q	93	1.48	Secondary metabolites biosynthesis, transport and catabolism
R	561	8.92	General function prediction only
S	350	5.56	Function unknown
-	2539	40.35	Not in COGs

## Insights from the genome sequence

Until now, only two genome sequences of the strain *F. pacifica* WPAGA1^T^ and *Flammeovirga* sp. OC4 were available within the genus *Flammeovirga* *.* Here, a whole genome comparison with these three strains have been done (Table 6). The genome of *Flammeovirga* sp. SJP92 is nearly 2 Mb bigger in size than *F. pacifica* WPAGA1^T^, but almost the same as *Flammeovirga* sp. OC4. The G + C content of *Flammeovirga* sp. SJP92 (34.8%) is slightly different with *F. pacifica* WPAGA1^T^ (33.8%) and *Flammeovirga* sp. OC4 (34.9%). The gene number of *Flammeovirga* sp. SJP92 is different from these two strains (6, 519 & 4, 857 & 5, 898).

**Table 6:** Comparison of genomes with *Flammeovirga* sp. SJP92, *F. pacifica* WPAGA1^T^ and *Flammeovirga* sp. OC4

Genome Name	*Flammeovirga* sp.SJP92	*F. pacifica* WPAGA1^T^	*Flammeovirga* sp.OC4
Genome size (bp)	8, 534, 834	6, 507, 364	8, 065, 497
Gene count	6, 519	4, 857	5, 898
Protein coding	6, 291	4, 739	5, 759
Protein with function	4, 240	4, 708	5, 596
Plasmid number	0	0	0
rRNA	5	3	2
tRNA	93	68	67
GC%	34.8	33.8	34.9
Contigs	123	131	214
CRISPR repeats	1	NA	5
Genes of agarase	13	10	5

Annotation of the genome indicated that this strain possessed many agarase (14 agarases at least), which was coincident with its high agar-degrading ability. Many sulfatases were also predicted and sequence alignment of proteins indicated that these sulfatases were novel. It is an aerobic strain and the existence of genes encoding superoxide dismutase and catalase were consistent with this phenotype. *Flammeovirga* sp. SJP92 contained many genes related to the metabolism and transport of amino acids. Also, metabolic pathway analysis and Biolog GN2 experiments illustrated that this strain could utilize many amino acids. These evidences may reflect its ability to grow by using proteinaceous media as the carbon and energy source.

## Conclusions

*Flammeovirga* sp. SJP92 is another strain with the genome sequence of the genus *Flammeovirga* together with *F. pacifica* WPAGA1^T^ and *Flammeovirga* sp. OC4. It is an agar-degrading bacterium with efficient agarose liquefying ability and had an extracellular agarase system containing 14 agarases at least. These genomic data will provide insights into the mechanisms of how these agarases cooperation to degrade agar or other polysaccharide.
